# Droga Heroica de Dreser x Sinfonia Eroica de Beethoven

**DOI:** 10.36660/abc.20201176

**Published:** 2020-12-01

**Authors:** Claudio Pinho

**Affiliations:** 1 Pontifícia Universidade Católica de Campinas CampinasSP Brasil Pontifícia Universidade Católica de Campinas, Campinas, SP – Brasil; 2 Clínica Pinho ValinhosSP Brasil Clínica Pinho, Valinhos, SP – Brasil

**Keywords:** Drogas Ilícitas, Heroina, Cocaina, Farmacologia, Eletrocardiografia/métodos, Analgésicos Opioides/uso terapêutico

Como amante da música clássica, frequento a sala São Paulo de Concertos com regularidade. Para lá chegar, obrigatoriamente passo por uma região de concentração de usuários de drogas chamada de cracolândia. Nesses momentos encontro de perto os importantes efeitos sociais e danosos da drogadição na saúde de seus usuários. No entanto, agora me deparo de um modo diferente através deste artigo “Heroin and Electrocardiography- Effect of Heroin on Electrocardigraphy parameters”^1^ que se soma a pequena literatura médica que procura acrescentar conhecimento aos efeitos cardíacos da heroína no coração e buscar sinais de sua ocorrência.

Os autores de “Heroin and Electrocardiography- Effect of Heroin on Electrocardigraphy parameters”^1^ deixam claro a pequena quantidade de informações sobre o assunto. Pesquisa no Pubmed mostra referência de 1980 intitulada “ECG examinations 115 heroin addicts”^2^ sem acesso ao seu conteúdo. Muito pouco encontramos desde então sobre este tema. Como explicar esse pouco interesse científico nesse interim sabendo que a drogadição cresce em números alarmantes. Fica aqui o primeiro questionamento: Pouco interesse dos pesquisadores nessa área? Pouca destinação de fomento financeiro para esta área? Dificuldades logísticas de pesquisas nesse campo? Desinteresse por esse grupo social?

A magnitude dos números é impactante, dados de 2019 da *World Drug Report* , publicação da *United Nations Office on Drugs and C* rime (UNODC) revelam que cerca de 35 milhões de pessoas tiveram alguma desordem em decorrência do uso de drogas sendo que apenas 1 em cada sete recebeu tratamento.

A dependência leva ao uso desenfreado com riscos de consequências letais. No caso da heroína a mortalidade gira em torno de 1% a 3% dos usuários, sendo mais frequentemente relatada como consequência de depressão respiratória.^3 , 4^ No entanto, agressão miocárdica e arritmias podem ocorrer e estarem subdiagnosticadas.^5 , 6^

Os autores de “Heroin and Electrocardiography- Effect of Heroin on Electrocardigraphy parameters”^1^mostram a ocorrência de alteração na repolarização ventricular dos usuários de heroína, mais precisamente diminuição do intervalo QT e QTc além da medida Tpe. Estas alterações foram encontradas nos eletrocardiogramas (ECGs) realizados em até 12 h após uso da heroína, tempo de ação de seu metabolito ativo a 6-acetilmorfina. Estas alterações encontradas poderiam sinalizar a predisposição de eventos arrítmicos nesse grupo levando a originar ações diagnósticas e terapêuticas voltadas aos distúrbios do ritmo e seus riscos.

Como a dependência a heroína é muito elevada e sua abstinência insuportável, o objetivo terapêutico sempre foi a troca de opioides procurando um menor dano. Com isso a metadona foi utilizada para contrabalançar os efeitos danosos da heroína. Isto fez com que o uso associado impedisse de conhecermos as ações cardíacas exclusivas da heroína. Também é relevante salientar que o uso mais frequente não é o inalatório e sim o endovenoso, que frequentemente está associado a adulterantes de ação cardiovascular, motivo pelo qual as ações cardíacas exclusivas da heroína eram desconhecidas em grande parte até agora. Os autores de “Heroin and Electrocardiography- Effect of Heroin on Electrocardigraphy parameters”^1^conseguiram isolar as ações exclusivas da heroína em parâmetros eletrocardiográficos da repolarização ventricular ao incluir no estudo pacientes em tratamento da drogadição que estavam utilizando a buprenorphine que é um opioide agonista parcial sem ações que interfiram no ECG. Com isso puderam comparar o ECG de 16 pacientes em uso de heroína antes e após a parada do seu uso e encontrar como consequência o aumento do QT e QTc com o afastamento da drogadição. Vale ressaltar que foram apenas 16 de 66 pacientes que finalizaram a proposta terapêutica para a drogadição de heroína, ou seja 24,24% mostrando a baixa adesão a esta terapia.

Os autores de “Heroin and Electrocardiography- Effect of Heroin on Electrocardigraphy parameters”^1^ agregaram conhecimento a este tema com elegância deixando claro que novos estudos necessitam ser feitos.

No campo da música erudita este ano comemoramos o marco de 250 anos do nascimento de Ludwig Van Beethoven, compositor da Sinfonia Eroica apresentada ao mundo em 1804, sendo que a partir de 1895 a “droga heroica” compartilhou o mesmo nome, que foi dado por Heinrich Dreser, pesquisador da empresa farmacêutica Friedrich Bayer & Co, fazendo com que o nome comercial adotado por esta droga quando lançada no mercado em 1898 fosse Heroína, até ser proibida em 1924.^7^ Quanto a Beethoven, nós continuamos ouvindo sua música até hoje.
