# Supplementary material for: Droga Heroica de Dreser x Sinfonia Eroica de Beethoven
Source: Arq Bras Cardiol. 2020 Dec 1;115(6):1142–3. [Article in Portuguese] doi: 10.36660/abc.20201176 (PMC8133724; doi:10.36660/abc.20201176)
Supplement: Translation

# Translation: Heroin by Dreser x Eroica by Beethoven

**Authors:** Claudio Pinho

**Affiliations:** 1 Brazil Pontifícia Universidade Católica de Campinas, Campinas, SP – Brazil; 2 Brazil Clínica Pinho, Valinhos, SP – Brazil

**Keywords:** Street Drugs, Heroin, Cocaine, Pharmacology, Electrocardiography/methods, Analgesics Opioid/therapeutic use

As a Classical Music lover, I go to the São Paulo Concert Hall frequently. On my way to get there it’s necessary to drive through an area with high density of addicts on streets, known as Crackland. In that moment I get to see closely the important negative social effects and damage of addiction in its users. Now I meet this issue in a different way, reading the paper: “Heroin and Electrocardiography- Effect of Heroin on Electrocardiography parameters”^1^that adds an incremental knowledge in the medical literature about heroin cardiac effects and searches for signals of when this occurs.

The authors of “Heroin and Electrocardiography- Effect of Heroin on Electrocardiography parameters”^1^make it clear that there is little scientific information published in this field. Research in the PubMed shows a 1980 reference called “ECG examinations 115 heroin addicts”^2^with no access to the abstract or the full-text contents. Very little was found about this specific topic since then. How can this be explained considering addiction has since then increased in alarming scale? Is there lack of interest by researchers in this area? Are there little financial resources for researchers? Logistical difficulties for studies in this field? Lack of interest in this social group?

The magnitude of the numbers is striking. Data from the “World Drug Report” published in 2019 by “The United Nations Office on Drugs and Crime (UNODC) reveals that 35 million people had some kind of disorder as a consequence of substance abuse and only one person in each seven received treatment.

Addiction leads to rampant use with risk of death. In the case of heroin, the risk of death is about 1-3% among the addicts and it is most frequently related to respiratory depression^3 , 4^although myocardial injury and arrhythmia can occur and may probably be underdiagnosed.^5 , 6^

The authors of “Heroin and Electrocardiography- Effect of Heroin on Electrocardiography parameters”^1^show an occurrence of abnormalities in the ventricular repolarization of heroin users, specifically decreased QT and QTc interval plus Tpe measure. These abnormalities were found in electrocardiograms performed in up to 12 hours after the use of heroin, the time of action of its active metabolit 6-acethilmorphine. These electrocardiograms abnormalities can be signalizing the predisposition to arrhythmic events in this group leading to therapeutic actions concerning to the rhythm disorders and the specific risks.

As the heroin dependence is high and its abstinence is unbearable, the therapeutic choice has always been the replacement by other less damaging opioids. In this way, methadone has been used as a substitute with less harmful effects than heroin. This fact has made it difficult to differentiate cardiac effects caused exclusively by heroin. It is also important to mention that the most frequent way of heroin use is intravenous and that many times it is mixed with adulterants which have cardiac effects. There are therefore multiple reasons why the exclusive cardiac actions of heroin were mostly unknown until now. The authors of “Heroin and Electrocardiography- Effect of Heroin on Electrocardiography parameters”^1^succeeded in isolating the heroin exclusive actions in ventricular repolarization electrocardiographic parameters by including in the study patients under treatment for addiction who were using buprenorphine, that is a partial agonist opioid that doesn’t change the parameters of repolarization in the electrocardiogram. In this manner, they could compare the electrocardiogram of 16 patients in the use of heroin before and after they stopped the drug and they were able to find an increase of duration of QT and QTc after discontinuing the addiction. It is important to say that only 16 from 66 (24,24%) patients followed the therapy until the end, showing the low adherence to the treatment.

The authors of “Heroin and Electrocardiography- Effect of Heroin on Electrocardiography parameters”^1^have given us, in an elegant way, a new contribution for the knowledge in this area and they have made it clear that other studies will be necessary to clarify this issue completely.

In the field of Classical Music, this year we are celebrating 250 years of Ludwig Van Beethoven’s birthday. He composed the famous “Eroica Symphony“ in 1804. In 1895, a German researcher named Heinrich Dreser working for the pharmaceutic industry Friedrich Bayer & Co became interested in the potential therapeutic benefits of the diacetylmorphine and called it the “heroic drug”. In 1898, the drug was launched in the market and it was called Heroin. This commercial name was adopted until 1924^7^when it was finally prohibited. And about Beethoven, we still keep on listening to his music until today.
